# The Influence of Physical Activity, Anxiety, Resilience and Engagement on the Optimism of Older Adults

**DOI:** 10.3390/ijerph17218284

**Published:** 2020-11-09

**Authors:** Alfonso Martínez-Moreno, Ricardo José Ibáñez-Pérez, Francisco Cavas-García F, Francisco Cano-Noguera

**Affiliations:** Department of Physical Activity and Sports, University of Murcia, 30720 Santiago de la Ribera-San Javier, Spain; ricardojose.ibanez@um.es (R.J.I.-P.); francisco.cavas@um.es (F.C.-G.F.); francisco.cano@um.es (F.C.-N.)

**Keywords:** older adults, aging, positivism, quality of life

## Abstract

The purpose of this study was to learn how physical activity, anxiety, resilience and engagement can influence optimism in older adults. An observational, quantitative, descriptive and transversal design was used with non-probabilistic sampling. A descriptive statistical analysis of the sample, Cronbach’s alpha test of internal consistency and linear correlation using Pearson’s correlation coefficient (*r*) were performed. In addition, a t-Student test, analysis of variance (ANOVA), Kolmogorov–Smirnov test of normality and Levene test of homogeneity, as well as a multivariate linear regression model, were conducted. Participants who had not engaged in physical activity showed an increased total anxiety and significantly greater decrease in concentration compared to those who had engaged in physical activity. The Revised Life Orientation Test (LOT-R), Utrecht Work Engagement Scale (UWES) and resilience of participants who had not engaged in physical activity were significantly lower than those of the participants who had engaged in physical activity. Those with a partner showed significantly lower decreases in concentration compared to single women. Regarding UWES, the current scores and dedication of couples were significantly higher than singles, as for resilience. In addition, the levels of pessimism in participants living on the coast were significantly higher compared to those living inland; in addition, a greater number of days with less anxiety is seen in those who performed physical activity. A multivariate linear regression model, *F*(7, 349) = 30.6, *p* < 0.001, explained 38% of the variance of LOT-R; those attending a public center had a lower LOT-R than those who did not, and high values of anxiety were associated with low levels of LOT-R, while high values of resilience were associated with high values of LOT-R. The results from the study provide support for future programs for older adults, in order to be able to determine in a much more precise way the objectives of programs intended for users of this age group.

## 1. Introduction

Today, the study of human behaviour in sport is an issue that has received considerable attention and is becoming increasingly important in the development of sports stakeholders, for coaches, teachers and managers alike. Thus, according to Gill et al. [1], it is necessary to understand psychological aspects related to sport, including the scientific study of people and their behaviors in the context of sport and physical activity as well as the practical applications of such knowledge. Eime et al. [2] directly linked sport engagement with physical health to an improvement in the psychological and social health of the individual.

The frequent practice of moderate-intensity Physical Activity (PA) has always been regarded as healthy, but it was not until the 1970s that physical activity began to be considered beneficial for both physical and mental health [3]. When the practice of PA is satisfactory for the subject, the subject judges his or her skill favorably and possesses a positive attitude towards the skill [4,5,6,7,8]. Research in physical activity and self-perception has suggested that attitudes to participation in physical sports activities increase feelings of competence and improve self-esteem more than more sedentary lifestyles [9].

In a study of older adults (OA) [10], it has been found that the practice of physical activity or exercise positively influenced both OAs’ physical health and psychological well-being [11,12]. OAs’ regular participation in physical activity programs led by a specialist was found to be an effective way to reduce or prevent some of the negative effects of aging [13,14]. Regular physical exercise adapted for OMs is the best non-pharmacological therapy for the main diseases associated with aging [15], so physical activity is a key element in the prevention of pathologies [16]. According to Graham et al. [17], physical activities such as basketball, tennis, weightlifting, self-defense and swimming helped participants improve and maintain their physical and mental health and quality of life. In addition, Nieman and Pedersen [18] reported that a moderate-intensity exercise program had a beneficial effect on the immune system. Specifically, moderate-intensity exercise was found to reduce the duration of illness. OAs have the same psychological and social needs to stay active as young people. Only when an individual performs physical activity does he or she feel happy, satisfied and adapted [19]. These effects manifest not only in the short term, but also in the long term, extending to over a year [20].

The theoretical pluralism characteristic of psychology has also contributed to complicating the task of establishing a single and valid definition of anxiety [21,22,23], especially if we consider that each psychological theory has developed its own explanatory model and its own definition. From a cognitive-behavioral perspective, Piqueras [24] conceptualized anxiety as cognitive and behavioral responses of an organism in response to a situation of physical or psychological threat or danger. Anxiety has been studied as part of other broader constructs, such as stress and fear of failure, but it has rarely been studied independently [25]. Anxiety manifestations can also be the product of age-specific health problems, or psychosocial changes within the family and socially. Its main impact is on capacity functionality, specifically on the development of everyday activities and quality of life [26]. The prevalence of anxiety disorders in old age is high enough to merit clinical care [27] but has not been given much attention in the literature [28]. Although it is highly common for older adults to complain about anxiety, its nature and clinical significance are not yet known [27]. Lasher and Faulkender [29] emphasized the importance of anxiety in aging as a major factor in the adjustment process in relation to old age.

Unlike anxiety, the role of resilience in the aging process has attracted much attention in the scientific community [30]. Current theories treat resilience as a multidimensional factor, including variables such as the ability to solve problems. In line with this, some authors have described it as the ability to face, resist and overcome adversity with more resources and better results than others [30] or as a dynamic process through which adaptive growth is achieved after effectively facing stressful situations, challenges and, above all, after overcoming adversities [31,32,33]. In addition, resilience has traditionally been associated with concepts such as adaptation [34]. Resilience has been found to have a direct impact on health, well-being and life satisfaction [35,36,37].

The results of studies such as that of Hosseini and Besharat [38] have shown that resilience is positively associated with sports success and psychological well-being, and has been negatively associated with stress, which often leads to a state of distress and non-adaptation. García-Gómez and Aldana [39] suggested that resilience could be either innate or acquired; although some people seem to possess some capacity for tolerance towards frustrations, difficulties or diseases from birth, it is also possible to learn such tolerance later in life by incorporating it into one’s personal repertoire of new ways of thinking and doing. Research in the field of senior citizens, such as that of Lavretsky [40] has promoted resilience as a strategy to prevent disorders in this advanced stage of life. Some research has determined the role of resilience as a predictor of well-being [41,42], and in a study by Meyers [43], high resilience was found to be a protective factor against anxiety, working positively for both the patient and the caregiver. However, overall, resilience has not received enough attention in the adult area [44].

It has recently been proposed that resilience is related to optimism [45,46]. Optimism is one of the most relevant constructs of positive psychology [47] and can be defined as the willingness to have positive expectations about what will happen in the future [48]. Optimism has been found to be beneficial for both physical and psychological well-being, while pessimism has been identified as a predisposing factor for depression [49,50]. Optimism increases the ability to socialize and build social support networks with friends and family [51]. Thus, optimists have skills with which to achieve their goals because they hope to achieve them, while pessimists understand that their results will always be negative. In addition, optimists have higher levels of health and well-being than pessimists [52,53,54]. Optimism has been described as a predictor of resilience in people who have been in highly traumatic situations [55].

Finally, commitment is a psychological factor that holds a salient role in society, whether in the personal or work sphere. Engagement can be understood as a sense of energy and a need to face up to what one is doing or practicing. It can be understood as the opposite of the term ‘burnout’. A more complete definition is that engagement is a positive, satisfying and work-related mental state, characterized by vigor, dedication and absorption. More than a specific and momentary state, engagement refers to a more persistent and influential affective-cognitive state, which is not focused on a particular object, event, individual or behaviour [56]. We know that optimism mediates anxiety [57], that resilience is a modulator of stress in general [58] and is related to optimism and commitment [59]. Based on all of the above, the following six objectives for the present research have been developed: (i) Determine the correlations between the anxiety, optimism, engagement and resilience scales; (ii) Identify the differences in the anxiety, optimism, engagement and resilience of participants who practiced physical activity and those who did not; (iii) Compare groups depending on marital status (married or single); (iv) Compare groups based on the participants’ place of residence (littoral or inland); (v) Compare groups based on occupation (retired, unemployed or working).(vi) Investigate the effects of variables (place of residence, type of center, physical activity) on engagement, anxiety and resilience in optimism.

## 2. Materials and Methods

For this research, an exploratory and descriptive methodology was chosen, which is usually used in research of this type. It is quantitative in nature, as this methodology is appropriate and suitable for addressing the objectives. The study is an ex-post facto study of a non-experimental and transversal type through a questionnaire. The sampling was non-probabilistic, since the researcher selected the sample based on certain subjective criteria and the nature of the research [60]. The fieldwork was carried out between October and November 2019.

### 2.1. Participants

The initial sample comprised 410 participants and was reduced to 381 participants after applying criteria to minimize biases of minimal effort (answers had the first box checked) and acquiescence (responding with an attitude, spontaneous and thoughtless, marking the same value in the scales). Of the final sample, 50.1% were male and 49.9% were female, aged between 52 and 94 years with an average age of 68.8 years (*SD* = 8.7). Older adults were understood as those over 52 years, since many of these were retired. In terms of marital status, 71.7% of subjects had a partner and 28.3% were single. With regard to education, 38.6% of the participants were not educated, 33.3% had completed primary education, 16.0% had a Bachelor’s/ Vocational training degree and 12.1% had completed a university degree. Most of the participants, 260 participants (68.2%), were retirees, and the remaining 25 (6.6%) were unemployed, 95 (24.9%) were working, and one was studying (0.3%). Regarding the center at which the study was performed, 30.2% of the participants attended a public center, 9.3% attended a private center and 60.6% did not attend either. Of the sample, 85% are institutionalized—live or work in a public/private/concert facility—and 15% are not. Of the participants, 71.1% claimed to do some form of physical activity, 28.9% indicated that they did not engage in any physical activity, and of the participants who were physically active, 8.3% exercised one day a week, 32.3% exercised two days a week, 16.5% exercised three days a week and 42.9% exercised more than three days a week. Regarding geographical situation, 38.1% lived on the coast and 61.9% lived inland.

### 2.2. Procedure

Participants were contacted directly on the street, at the entrance and exit of the centers, as well as by contacting the heads/directors/managers of the different institutions (senior centers/social centers/sports centers) and administrations that included OA among their members, depending on place of residence. The design did not contain any ethical aspects requiring prior authorization from the Bioethics Committee of the University of Murcia, Spain. After explaining the objectives, those who had decided to participate in the study completed the informed consent form and questionnaire in the presence of one of the researchers trained for this purpose. The protocol was applied respecting at all times the Helsinki 2013 declaration, thus ensuring that all participants received the same information when completing the questionnaires. The study complied with European Data Protection Law.

### 2.3. Instrument

The instrument in this research was a questionnaire consisting of several tests: the Sport Anxiety Scale-2, SAS-2 Anxiety questionnaire, the Revised Life Orientation Test (LOT-R) test, the Utrecht Work Engagement Scale (UWES), and the Connor-Davison Resilience Scale CD-RISC questionnaire. The SAS-2 Anxiety Questionnaire [61] contains a total of 15 items measuring three dimensions: somatic (A-S), concern (A-P) and deconcentration (A-D) on a 4-point Likert scale. The total score is based on the sum of all of the items and has a range of values between 15 and 60. In this study’s sample, the questionnaire showed good internal consistency (⍺ = 0.88) as a whole and for both the A-P (⍺ = 0.83) and A-D (⍺ = 0.81) dimensions. Only the A-S dimension showed fair reliability (⍺ = 0.79).

For the measurement of optimism in the questionnaire, the LOT-R test was used. This determines the participants’ expectations for positive or negative results from events that may occur. For the purposes of this study, the Spanish version of the test by Otero et al. [62] was used. Composed of 10 items measuring two dimensions, optimism (O) and pessimism (P), responses were recorded on a 5-point Likert scale. In the sample under study, the reliability of the total scale was ⍺ = 0.86 and divided by dimension; the optimistic dimension was ⍺ = 0.84 and the pessimism dimension was ⍺ = 0.85.

To measure the level of engagement (UWES) [63], consisting of 9 items and 3 dimensions, was used. The dimensions included: vigor (E-V), dedication (E-D) and absorption (E-A). Each response was recorded on a 7-point Likert scale, ranging from 0 (never) to 6 (always). The total engagement value was obtained by adding the scores from all of the items and dividing the sum by the number of dimensions. The average scores of the three dimensions were obtained by adding the scores of each subscale and dividing the result by the number of items in the respective subscale. Thus, the UWES-9 could yield three sub-scores, corresponding to each subscale, and a total score within the range of 0 to 6 points. In the sample analysed, the questionnaire presented a reliability of ⍺ = 0.85 for the total scale. In the analysed sample, the UWES questionnaire presents good internal consistency of ⍺ = 0.85 as a whole and for both the vigor dimension (⍺ = 0.82) and the absorption dimension (⍺ = 0.80). Only the dedication dimension showed an acceptable reliability (⍺ = 0.79).

Resilience was evaluated using the short version of the CD-RISC in Spanish [64]. This consisted of 10 items corresponding to the original scale from Connor & Davidson [31]. Responses were given on a 5-point Likert scale ranging from 0 (totally disagree) to 4 (totally agreed). In the sample analysed, the CD-RISC questionnaire has a reliability of ⍺ = 0.84.

### 2.4. Statistical Analysis

For the descriptive statistical analysis of the sample, the number of cases present in each category and the corresponding percentages for the qualitative variables were obtained. For the quantitative variables, the minimum, maximum, mean, and standard deviation (SD) values were used. Cronbach’s alpha was calculated with the aim of checking the reliability of the different scales in this particular sample, and correlations between variables were calculated using the Pearson linear correlation coefficient (*r*). In addition, with regard to quantitative variables, the t-Student test was performed for means comparisons between two groups, and for comparisons of more than two groups an analysis of variance (ANOVA) was performed. The assumptions of normality and uniformity of variances necessary for the means comparisons were tested using the Kolmogorov-Smirnov test and the Levene test, respectively. Finally, a multivariate linear regression model was produced to determine the possible effects of the demographic variables (i.e., place of residence, type of center and physical activity) as well as the UWES, SAS-2 and LOT-R scales. The statistical analyses were performed on the SPSS 25.0 program for Windows (IBM, New York, NY, USA). Statistical significance was defined as *p* < 0.05.

## 3. Results

In this section, the data obtained in the research will be explained. To facilitate the understanding of the results obtained, the details have been provided in Table 1, Table 2, Table 3 and Table 4.

The mean (typical deviations), Cronbach’s alpha reliability indices and correlations between the different scales used in the research are shown in Table 1. The internal consistency indices were all greater than 0.70, indicating high reliability.

The SAS-2 scale correlated statistically significantly and positively with A-S, A-P, A-D and P and negatively with LOT-R, O, UWES, E-V, E-D, E-A and Resilience. A-S correlated significantly and positively with A-P, A-D and P and negatively with the remainder of the scales and dimensions. A-P correlated statistically significantly and positively with A-D and P and negatively with LOT-R, O, UWES, E-V and Resilience. It did not correlate with the remaining dimensions. A-D correlated statistically significantly and positively with P and negatively with the remainder of the scales and dimensions. The LOT-R correlated significantly and negatively with P and positively with the remaining scales and dimensions. O correlated significantly and negatively with P and positively with the other scales and variables. P only showed a significant negative correlation with Resilience. The UWES correlated significantly and positively with all of the scales and dimensions, as well as with E-V, E-D, E-A and Resilience.

The results of each scale according to Physical Activity, as well as the results of the t-Student tests performed to identify statistically significant differences between those who did not perform physical activity and those who did, showed statistically significant differences (*p* < 0.001) overall on the SAS-2, LOT-R, UWES, and CD-RISC scales. In relation to anxiety, people who do not engage in physical activity had significantly higher total levels of anxiety (*M* = 36.65, *SD =* 10.30), as well as in the A-S (*M* = 11.46, *SD =* −4.63) and A-D (*M =* 11.70, *SD =* 4.69) subscales, compared to those engaged in physical activity (*p* < 0.001). The total LOT-R and O of those who did not exercise was also significantly lower than that of those who did (*p* < 0.001). In terms of UWES, all of the scores of people who did not perform physical activity were significantly lower than those of the participants who did perform physical activity (*p* < 0.001). Finally, the resilience scores of non- physical activity participants were significantly lower than the resilience scores those who performed physical activity (*p* < 0.001).

With regard to the number of days performing physical activity, Pearson’s correlation coefficient showed a statistically significant negative relationship between anxiety and physical activity (*r* = −0.274, *p* = 0.004), with reduced anxiety as the number of days increased. The other scales did not present any significant relationships with the number of days engaged in physical activity.

Marital status showed statistically significant differences in the SAS-2 (*p* = 0.085), LOT-R (*p* = 0.365), UWES (*p* = 0.072) and Resilience (*p* = 0.002) scales. With regard to anxiety, those with partners showed an A-D significantly lower than that in single women (*p* = 0.027). The UWES data revealed that the scores in E-V (*M* = 4.32, *SD =* 11.32) and E-D (*M =* 4.39, *SD =* 11.28) of those with a partner were significantly higher than the scores for single women, *p* = 0.038 and *p* = 0.044, respectively. Lastly, the resilience scores for those with a partner (*M* = 28.31, *SD =* 7.77) were significantly higher than the single participants’ scores (*p* = 0.002).

Depending on the place of residence, coastal or inland, the results showed statistically significant differences in P (*p* = 0.0.29) in the LOT-R scale, where the scores of those residing on the coast (*M* = 6.55, *SD* = 2.46) were significantly higher than for those living inland (*M* = 5.93, *SD* = 2.80).

In terms of occupation, the ANOVA showed statistically significant differences in the anxiety, optimism and engagement scales. Retirees has significantly higher scores in total anxiety and deconcentration than the unemployed and workers, with no significant differences between the participants who were unemployed and those who worked. Workers also had significantly higher scores than retirees and the unemployed with regard to optimism, with no significant difference between retirees and the unemployed. The total UWES scores and the scores on the E-V and E-D dimensions were significantly lower in the unemployed participants than in the retirees and workers, with no significant difference between the retirees and workers. The remaining scales did not show any significant differences.

In terms of age groups in Table 3, the ANOVA showed statistically significant differences in the scales of anxiety, commitment to optimism and resilience. In relation to anxiety, adults <55 years showed the highest values for total anxiety and worry and adults >75 years for somatic anxiety and distractibility. With regard to age groups, there are significant differences between the groups of <55 and >75 years in relation to those of 55–64 and 65–75 years in total anxiety and somatic anxiety. In terms of deconcentration, there are significant differences between the >75 group and the other three groups (<55; 55–64 and 65–75 years).

Adults aged 55–64 years achieve the highest scores in total LOT and optimism, while in pessimism it is the older adults <55 years who obtain the highest score. In the LOT there are significant differences between the <55 years and the other age groups in optimism and pessimism.

In the UWES there are significant differences between adults >75 years and the other three age groups (<55; 55–64 and 65–75 years) in UWES total, vigor, dedication and absorption.

In terms of resilience there are significant differences between <55 and >75 years in relation to adults over 55–64 and 65–75 years.

When comparing the different scales in relation to sex, no significant differences were found in any of the scales or in their dimensions.

To determine the possible effect of the variables, place of residence, type of center and PA practice or not, as well as the UWES, Anxiety, Resilience and LOT-R scales, a multivariate linear regression model was carried out, the results of which are shown in Table 4. The model was statistically significant, F (7,349) = 30.6, *p* < 0.001, explaining 38% of the variability of optimism. Of the demographic variables, the type of center showed a statistically significant effect, with those attending public centers having lower LOT-than those who do not belong to any facility. In terms of scales, anxiety and resilience showed significant effects, such that higher values in anxiety were associated with lower levels of LOT-R, while higher values in resilience were associated with higher values in LOT-R. The assumptions of normality, independence and homoscedasticity are verified after the analysis of waste, after carrying out Pearson’s correlation between the explanatory variables, the residues (r ≈ 0) being the exogenous variables.

**Figure 1:**
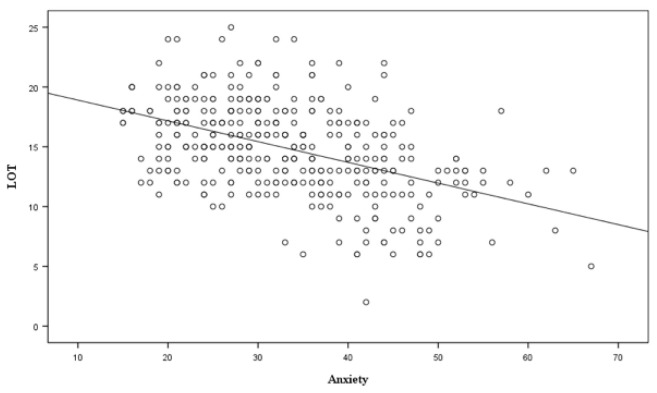
Relationship between anxiety and optimism (Source: the Authors).

**Figure 2:**
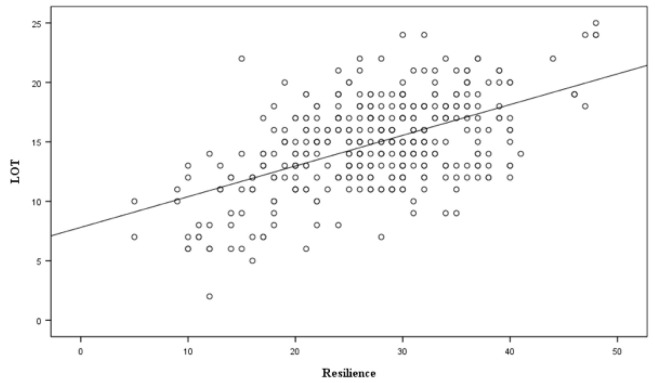
Relationship between resilience and optimism (Source: the Authors).

Attached are Figure 1 and Figure 2 where we can see the results of each scale with respect to optimism.

## 4. Discussion

The benefits of physical activity on OA’s physical and psychological states, as well as on satisfaction with OA life, are undeniable [3,65]. It is thus necessary to be able to specify what these benefits are and to narrow the extent to which these aspects affect OAs or not. To this end, the study aimed to determine the correlations between anxiety, optimism, engagement and resilience. It was revealed that there was a negative correlation between anxiety and resilience, coinciding with Perdomo [66], as well as between anxiety and optimism, in line with Suarez’s study [67]. In addition, the data determined a significant and positive correlation between engagement and resilience, which was partly similar to the findings of Gil et al. [68]. Equally significant was the inverse correlation between anxiety and resilience, coinciding with García-León et al. [69]. The data showed that participants with higher resilience had lower levels of anxiety, in line with Benetti and Kambouropoulos [70] and Beutel et al. [71], considering resilience as a component of adequate social adaptation, which is associated with mental health [72]. On the other hand, it was observed that optimism was positively related to resilience as Maury-Ortiz et al. [73] also found.

Another objective of the study was to identify the differences between participants who practiced physical activity and those who did not practice physical activity. The results showed that the participants in the study who did not practice physical activity had a total anxiety, A-S and A-D, significantly higher than those who performed physical activity. This result was in the line with other research that had studied these relationships [74,75,76]. In relation to optimism the data showed a clearly significant negative relationship between non-physical activity participants and optimism, similar to that of previous studies [77,78], indicating that optimism could be a protective factor against anxiety [79]. 

A third objective was to study the different scales in relation to whether there were differences depending on marital status. The results showed statistically significant differences in all of the scales. In terms of anxiety, those with a partner showed significantly lower deconcentration compared to single women, in line with similar studies on psychological well-being [80]. With regard to engagement, the current scores and dedication of couples were significantly higher than in single women, consistent with Jürschik’s study [81], where living situation was found to be only one of the factors influencing the deterioration in OA. Finally, the resilience score of couples was significantly higher than in single women, in line with the studies of Losada and Alvarez [82], which studied depressive symptoms, although this result differs from Cruz’s study [83], where no significant differences were found between resilience and living alone or with a partner in a sample of institutionalized OA [84].

With regard to the third objective, place of residence (coastal or inland) showed statistically significant differences only in pessimism on the LOT-R scale, where the score of coastal residents was significantly higher than those living inland. Caicedo and Berbesi [84] also had similar findings, with differences in self-referred health in different geographical areas. This study, however, did not differentiate between coastal and inland residences. Finally, the results showed that more physical activity practice days produced less anxiety, coinciding with [85].

In exploring the differences in occupation and the different scales, the results showed statistically significant differences in anxiety, optimism, and engagement. In total anxiety and in deconcentration, retirees showed a significantly higher score than the unemployed and workers, with no significant difference between the unemployed and workers. In total optimism, workers had a significantly higher score than retirees and the unemployed, with no significant difference between the latter two groups. In engagement, the total score and the strength and dedication of the unemployed were significantly lower than those of retirees and workers, without significant differences between retirees and workers. This possibly indicates that OAs, both retired and working, carry out a successful and positive aging process, linked in the scientific literature to the health and well-being of OAs [86].

The final objective was to show the possible effects of demographic variables (place of residence, type of center and Physical Activity) and the anxiety, engagement and resilience scales on the LOT-R. Of the demographic variables, the type of center showed a statistically significant effect so that people attending a public center had a lower LOT-R score than those who did not attend any facility. In terms of the scales, anxiety and resilience showed significant effects such that higher values of anxiety were associated with lower levels of optimism, while higher values of resilience were associated with higher values in LOT-R, coinciding with the results described in the previous scientific literature [45,46] and indicating Vizoso’s findings [87] that optimism has predictive power over resilience, corroborating previous studies [35,36,37].

The study had some limitations, given the cross-cutting nature of the data. The first limitation is related to the impossibility of establishing generalizations. In addition, causal inferences of the results could not be obtained. Finally, it is interesting to propose for future research the establishment of other conditions that may have causal effects on optimism against other psychological variables, as well as the extension of this study to different cultural and geographical contexts, adding socioeconomic variables that better contextualize the specific needs of OAs.

## 5. Conclusions

The data from the study was in line with previous studies. However, this work presented an important path in the study of the interrelationship between different psychological variables in this age group, with the many problems associated with it that, in one way or another, can impair the ability to develop a successful and positive aging process. Knowing these interrelationships may help to increase the quality of life for these OAs. The data in the study served as a support for future programs for OAs, in order to be able to determine in a much more precise way the objectives of the programs intended for users of this age group. 

Once the results were obtained and analysed, they were compared and analysed with other research in order to obtain precise conclusions on the analysed constructs and objectives raised, as shown in the previous section. 

Therefore, the OAs in the sample who practice physical activity on the greatest number of days, live in pairs and reside in the interior show low anxiety and high engagement values. The practice of physical activity is shown as a counterbalance to anxiety, optimism, commitment and resilience in the OAs of the sample. Participants attending a public center had lower LOT-R levels than those not attending a center. In terms of the scales, anxiety and resilience showed significant effects, so higher values of anxiety were associated with lower levels of LOT-R, while higher values of resilience were associated with higher values of LOT-R. 

In relation to anxiety, adults <55 and >75 show significant differences in relation to the age groups 55–64 and 65–75 in total anxiety and somatic anxiety, while in relation to deconcentration, adults <75 show significant differences in relation to the other age groups.

The OCs of <55 years show significant differences in optimism and pessimism on the LOT scale, as compared to the other age groups.

With regard to the UWES-9 scale, adults >75 present significant differences with respect to the other age groups in the total of the UWES-9 scale as well as in its dimensions.

The resilience scale indicates that there are significant differences between the groups of older adults <55 and >75 in relation to the groups of 55–64 and 65–75 years. There are no significant differences in worry in the anxiety scale and total LOT, with respect to the age groups. As a final conclusion, it should be noted that physical activity has a moderating effect on anxiety, and those who practiced more days of physical activity obtained lower values on the anxiety scale. Resilience acts as a catalyst for optimism; the greater the resistance, the greater the optimism and the lower the anxiety; and the lesser the optimism, the greater the anxiety.

## Figures and Tables

**Table 1:** Means, standard deviations, reliability and correlations of the scales.

	Mean (SD)	Cronbach’s alpha	1	2	3	4	5	6	7	8	9	10	11
Anxiety (SAS-2)													
1. Total	33.71 (10.48)	0.880	1										
2. Somatic (A-S)	9.88 (4.31)	0.797	0.865 *	1									
3. Concern (A-P)	13.16 (4.04)	0.839	0.717 *	0.408 *	1								
4. Deconcentration (A-D)	10.7 (4.52)	0.811	0.861 *	0.696 *	0.388 *	1							
Optimism (LOT-R)													
5. Total	14.85 (3.82)	0.860	−0.462 *	−0.447 *	−0.179 *	−0.483 *	1						
6. Optimism (O)	8.82 (2.55)	0.848	−0.353 *	−0.365 *	−0.154 *	−0.343 *	0.780 *	1					
7. Pessimism (P)	6.17 (2.69)	0.850	0.338 *	0.300 *	0.166 *	0.351 *	−0.783 *	−0.148 *	1				
Engagement (UWES)													
8. Total	4.27 (1.25)	0.852	−0.382 *	−0.404 *	−0.102 *	−0.423 *	0.409 *	0.547 *	−0.034	1			
9. Vigor (E-V)	4.23 (1.38)	0.821	−0.372 *	−0.379 *	−0.117 *	−0.409 *	0.417 *	0.530 *	−0.059	0.940 *	1		
10. Dedication (E-D)	4.3 (1.34)	0.790	−0.331 *	−0.363 *	−00.074	−0.367 *	0.381 *	0.519 *	−0.015	0.944 *	0.843 *	1	
11. Absorption (E-A)	4.27 (1.29)	0.806	−0.369 *	−0.392 *	−00.094	−0.410 *	0.346 *	0.484 *	−0.019	0.920 *	0.785 *	0.804 *	1
CD-RISC (Resilience)	27.49 (8.23)	0.844	−0.369 *	−0.365 *	−0.138 *	−0.392 *	0.542 *	0.639 *	−0.150 *	0.633 *	0.588 *	0.601 *	0.588 *

* *p* < 0.001.

**Table 2:** Descriptive and comparative analysis of the scores on the scales according to occupation.

	Occupancy, Mean (SD)			ANOVA		eta^2^
	Retired	Unemployed	Working	F (2, 377)	*p*-Value	
Anxiety (SAS-2)						
Total	34.84 (10.65) a	31.08 (10.87) b	31.39 (9.46) b	4.723	0.009	0.024
Somatic	10.15 (4.35)	8.83 (3.61)	9.45 (4.31)	1.68	0.188	0.009
Concern	13.29 (4.01)	12.17 (4.02)	13.09 (4.13)	0.864	0.422	0.005
Deconcentration	11.43 (4.64) a	10.32 (4.61) b	8.83 (3.58) b	12.116	< 0.001	0.061
Lot						
Total	14.54 (3.78)	14.48 (3.69)	15.76 (3.89) b	3.629	0.028	0.02
Optimism	8.78 (2).51)	8.32 (2.90)	9.07 (2.57)	0.986	0.374	0.005
Pessimism	6.43 (2.70)	5.84 (2).03)	5.52 (2.76)	4.307	0.014	0.022
Utrecht Work Engagement Scale (UWES)						
Total	4.21 (1.34) a	3.89 (1.29) b	4.53 (0.91)	3.645	0.027	0.019
Force	4.14 (1.48) a	3.92 (1.38) b	4.54 (1.02) a	3.544	0.03	0.018
Dedication	4.23 (1.44)	3.91 (1.32) b	4.60 (1.01) a	3.734	0.025	0.019
Absorption	4.24 (1.36)	3.84 (1.28)	4.47 (1.03)	2.573	0.078	0.013
Resilience	27.29 (8.62)	25.16 (9.13)	28.67 (6.70)	2.066	0.128	0.011

SD: standard deviation. eta^2^: effect size. a-b: two-to-two column comparisons. Between two different columns letters indicate statistically significant differences (Bonferroni correction).

**Table 3:** Descriptive and comparative scale scores according to age.

	Age, Mean (SD)				ANOVA		eta^2^
	<55	55–64	65–75	>75	*F (2, 377)*	*p*-Value	
Anxiety (SAS-2)							
Total	38.00 (8.56) a	31.66 (10.26) b	33.38 (9.90) b	37.22 (11.51) a	4.929	0.002	0.038
Somatic	10.86 (5.30) a	9.59 (4.27) b	9.32 (3.84) b	11.52 (4.96) a	5.159	0.002	0.04
Concern	16.29 (3.50)	12.84 (4.34)	13.15 (3.92)	13.46 (3.81)	1.783	0.15	0.014
Deconcentration	10.86 (4.88) a	9.26 (3.94) a	10.90 (4.38) a	12.34 (5.09) b	7.655	< 0.001	0.058
Lot							
Total	13.40 (4.51)	15.46 (3.37)	14.86 (3.70)	13.97 (4.60)	2.441	0.064	0.02
Optimism	7.71 (4.15) a	9.04 (2.21) b	9.00 (2.42) b	8.17 (3.05) b	2.732	0.044	0.021
Pessimism	9.14 (3.98) a	5.64 (2.44) b	6.21 (2.74) b	6.53 (2.60) b	4.951	0.002	0.038
UWES							
Total	4.44 (0.73) a	4.45 (1.00) a	4.41 (1.24) a	3.68 (1.44) b	7.742	< 0.001	0.058
Force	4.19 (0.81) a	4.46 (1.08) a	4.35 (1.39) a	3.64 (1.60) b	6.623	< 0.001	0.05
Dedication	4.38 (0.95) a	4.51 (1.07) a	4.43 (1.35) a	3.71 (1.52) b	6.743	< 0.001	0.051
Absorption	4.76 (0.96) a	4.37 (1.11) a	4.45 (1.22) a	3.69 (1.51) b	7.315	< 0.001	0.055
Resilience	24.86 (5.76) a	28.58 (7.06) b	28.38 (8.21) b	24.12 (9.23) a	6.16	< 0.001	0.047

SD: standard deviation. eta^2^: effect size. a-b: two-to-two column comparisons. Between two different columns, letters indicate statistically significant differences (Bonferroni correction).

**Table 4:** Effects of variables and scales on Revised Life Orientation Test (LOT-R) scores.

	B (ET)	Beta	T	*p*-Value
Residence (littoral or inland)	−0.30 (0.35)	−039	−0.862	0.389
Center type				
None	Ref.			
Private	−0.10 (0.60)	−0.008	−0.174	0.862
Public	−0.85 (0.37)	−0.102	−2.282	0.023
Physical activity (Yes vs. No)	0.47 (0.38)	0.056	1.241	0.215
UWES	0.17 (0.18)	0.056	0.976	0.33
Anxiety	−0.10 (0.02)	−0.272	−5.73	< 0.001
Resilience	0.19 (0.03)	0.398	7.211	< 0.001
Assumptions				
Normality ^†^	*p* = 0.200			
Independence ^‡^	2.031			
Homoscedasticity ^+^	*p* = 0.721			

B: non-standardized regression coefficient. ET: typical error. Beta: standardized regression coefficient. ^†^ Test of Kolmogorov-Smirnov normality of waste. ^‡^ Test of Durbin-Whatson. ^+^ Levene test between residual and predicted values.

## References

[1] Gi Gill D., Williams L., Reifsteck E. (2017). Psychological Dynamics of Sport and Exercise.

[2] Eime R.M., Young J.A., Harvey J.T., Charity M.J., Payne W.R. (2013). A systematic review of the psychological and social benefits of participation in sport for children and adolescents: Informing development of a conceptual model of health through sport. Int. J.Behav. Nutr. Phys. Activ..

[3] Barriopedro M.I., Eraña I., Mallol L.L. (2001). Relación de la actividad física con la depresión y satisfacción con la vida en la tercera edad. Rev. Psicol. Dep..

[4] Crocker P., Eklund R., Kowalski K. (2000). Children’s physical activity and physical self-perceptions. J. Sports Sci..

[5] Hellín P., Moreno J.A., Rodríguez P.L. (2006). Relación de la competencia motriz percibida con la práctica físico-deportiva. Scand. J. Med. Sci. Sports.

[6] Lindwall L.M., Hassmen P. (2004). The role of exercise and gender for physical self-perceptions and importance ratings in swedish university students. Scand. J. Med. Sci. Sports.

[7] Ommundsen Y. (2003). Implicity theories of ability and self-regulation strategies in physical education classes. Educ. Psychol..

[8] Panter J., Jones A., Van Sluijs E., Griffin S. (2010). Attitudes, social support and environmental perceptions as predictors of active commuting behaviour in school children. J. Epidemiol. Commu. Health.

[9] Westerberg-Jacobson J., Edlund B., Ghaderi A. (2010). A 5-year longitudinal study of the relationship between the wish to be thinner, lifestyle behaviour and disturbed eating in 9–20 year old girls. Eur. Eat. Disord. Rev..

[10] Arraga-Barrios M., Sánchez-Villaroel M. (2007). Recreación y calidad de vida en adultos mayores que viven en instituciones geriátricas y en sus hogares. Un estudio comparativo. Espac. Abierto. Cuad. Venezol. Soc..

[11] Armadans I., Pérez A., Franco N. (1998). Actividad deportiva recreativa y tercera edad: Algunos criterios de gestión para potenciar su demanda. Rev. Multidiscipl. Gerontol..

[12] Rejeski W., Brawley L., Shumaker S. (1996). Physical activity and sedentary behaviour: A population-bases study of barriers, enjoyment and preference. Health Psychol..

[13] Méndez A., Fernández J. (2005). Prescripción de la actividad física en personas mayores: Recomendaciones actuales. Revista Española de Educación Física y Deportes.

[14] Mora J., González J.L., Mora M. (2005). Efectos de un programa de ejercicios sobre el gasto cardiaco en una población de mujeres adultas sedentarias. Revista Española de Educación Física y Deportes.

[15] Weisser B., Preuss M., Predel H.G. (2009). Physical activity for prevention and therapy of internal diseases in the elderly. Med. Klin. (Munich, Germany: 1983).

[16] Zurita F. (2015). Influencia de Los Factores Psicológicos Sobre las Lesiones Deportivas en Deportes de Equipo. Ph.D. Thesis.

[17] Graham G., Holt/Hale S.A., Parker M. (1998). Children Moving.

[18] Nieman D.C., Pedersen B.K. (1999). Exercise and immune function: Recent developments. Phys. Exerc. Med..

[19] Muñoz J. (2002). Psicología del Envejecimiento.

[20] Elavsky S., McAuley E., Motl R., Konopack J., Márquez D., Hu L., Jerome G., Diener E. (2005). Physical activity enhances long-term quality of life in older adults. Efficacy, esteem and affective influences. Ann. Behav. Med..

[21] Catell R.B., Scheier I.H. (1958). The nature of anxiety: Areview of thrite en multivariate analysis comprissing 814 variable. Psychol. Rep..

[22] Spielberger C.D. (1996). Anxiety and Behavior.

[23] Tyrer P.J. (1985). Estados de Ansiedad in Paykel, E.: Psicopatologia de los Transtornos Afectivos.

[24] Piqueras R.J., Martínez G.A., Ramos L.V., Rivero B.R., García L.L., Oblitas G.L. (2008). Ansiedad, depresión y salud. Suma Psicol..

[25] Stöber J., Pekrun R. (2004). Advances in test anxiety research. Anxiety Stress Coping.

[26] Korc E.M. (2005). Vivienda Saludable: Enlace entre la Investigación y las Políticas Públicas. Organización Panamericana de la Salud.

[27] Ownby R., Harwodd D., Arker W., Duara R. (2000). Predictors of anxiety in patiens with Alzheimer’s disease. Depress. Anxiety.

[28] Snyder A., Stanley M., Novy D., Averill P., Beck J. (2000). Measures of depression in older adults with generalized anxiety disorder: A psychometric evaluation. Depress. Anxiety.

[29] Lasher P.K., Faulkender P.J. (1993). Measurement of aging anxiety: Development of the Anxiety about Aging Scale. Int. Aging Hum. Dev..

[30] Schiera A. (2005). Uso y abuso del concepto de resiliencia. Revista de Investigación en Psicología.

[31] Connor K.M., Davidson J.R. (2003). Development of a new resilience scale: The ConnorDavidson Resilience Scale (CD-RISC). Depress. Anxiety.

[32] Masten A.S. (2018). Resilience theory and research on children and families: Past, present, and promise. J. Family Theory Rev..

[33] Rutter M. (2007). cResilience, competence, and coping. Child Abuse Neglect.

[34] Becoña E. (2006). Resiliencia: Definición, características y utilidad del concepto. Revista de Psicopatología y Psicología Clínica.

[35] Arrogante O. (2014). Mediación de la resiliencia entre burnout y salud en el personal de Enfermería. Enfermería Clínica.

[36] Hu T., Zhang D., Wang J. (2015). A meta-analysis of the trait resilience and mental health. Pers. Individ. Differ..

[37] Diener E.D., Emmons R.A., Larsen R.J., Griffin S. (2014). A Correlational Study on dispositional resilience, psychological well-being, and coping strategies in university students. Am. J. Educ. Res..

[38] Hosseini S.A., Besharat M.A. (2010). Relation of resilience whit sport achievement and mental health in a sample of athletes. Procdeia Soc. Behav. Sci..

[39] García-Gómez L., Aldana G. (2011). Voces infantiles en torno a la resiliencia: Las experiencias vitales de niños habitantes de una casa hogar en Ecatepec, estado de México. Uaricha. Revista de Psicología.

[40] Lavretsky H. (2012). Resilience, stress and mood disorders in old age. Ann. Rev. Gerontol. Greiatr..

[41] Rowe J.W., Kahn R.L. (2000). Successful aging and disease prevention. Adv. Renal Replac. Therap..

[42] Wagnild G. (2003). Resilience and successful aging: Comparison among low and high income older adults. J. Gerontol. Nurs..

[43] Meyers E.E., Presciutti A., Shaffer K.M., Gates M., Lin A., Rosand J., Vranceanu A.-M. (2020). The Impact of Resilience Factors and Anxiety During Hospital Admission on Longitudinal Anxiety Among Dyads of Neurocritical Care Patients Without Major Cognitive Impairment and Their Family Caregivers. Neurocrit. Care.

[44] Zurita F., Espejo T., Cofré C., Martínez A., Castro M., Chacón R. (2016). Influencia de la actividad física sobre la resiliencia en adultos con dolor de hombro. SPORT TK-Revista EuroAmericana de Ciencias del Deporte.

[45] Martínez-Martí M.L., Ruch W. (2017). Character strengths predict resilience over and above positive affect, self-efficacy, optimism, social support, self-esteem, and life satisfaction. J. Posit. Psychol..

[46] Ramírez-Fernández E., Ortega-Martínez A.R., Calero-García M.J. (2018). El optimismo como mediador entre la resiliencia y estados afectivos en adultos mayores. Estudios de Psicología.

[47] Marín E., Ortín F.J., Garcés de los Fayos E., Tutte V. (2013). Bibliometric analysis of burnout and optimism in sport. Revista Euroamericana de Ciencias del Deporte.

[48] Carver C.S., Scheier M. (2014). Dispositional optimism. Trends Cognitive Sci..

[49] Hart S.L., Vella L., Mohr D.C. (2008). Relationships among depressive symptoms, benefit-finding, optimism, and positive affect in multiple sclerosis patients after psychotherapy for depression. Health Psychol..

[50] Hirsch J., Wolford K., LaLonde S., Brunk L., Parker Morris A. (2007). Dispositional optimism as a moderator of the relationship between negative life events and suicide ideation and attempts. Cogn. Ther. Res..

[51] Díaz A., Ponsoda J.M., Beleña A. (2020). Optimism as a key to improving mental health in family caregivers of people living with Alzheimer’s disease. Aging Ment. Health.

[52] Extremera N., Durán A., Rey L. (2007). Perceived emotional intelligence and dispositional optimism–pessimism: Analyzing their role in predicting psychological adjustment among adolescents. Pers. Individ. Differ..

[53] He F., Cao R., Feng Z., Guan H., Peng J. (2013). The impacts of dispositional optimism and psychological resilience on the subjective well-being of burn patients: A Structural Equation Modelling Analysis. PLoS ONE.

[54] Segerstrom S.C., Carver C.S., Scheier M.F., Robinson M.D., Eid M. (2017). Optimism. The Happy Mind Cognitive Contributions to Well-Being.

[55] Segovia F., Moore J.L., Linnville S.E., Hoyt R.E., Hain R.E. (2012). Optimism predicts resilience in repatriated prisoners of war: A 37-year longitudinal study. J. Trauma. Stress.

[56] Schaufeli W.B., Salanova M., Gonzalez-Roma V., Bakker A.B. (2002). The measurement of engagement and burnout and: A confirmative analytic approach. J. Happiness Stud..

[57] Gutiérrez-Carmona A., Mondaca C.A., Urzúa A., Wlodarczyk A. (2020). Puede el optimismo mediar el efecto negativo de la ansiedad rasgo sobre el bienestar psicológico?. Interam. J. Psychol..

[58] Navarro-Abal Y., López-López M.J., Climent-Rodríguez J.A. (2018). Engagement (compromiso), resiliencia y empatía en auxiliares de enfermería. Enfermería Clínica.

[59] Vizoso C.M., Arias-Gundín O.A. (2019). Relación entre resiliencia, optimismo y engagement en futuros educadores. IJERI Int. J. Educ. Res. Innov..

[60] Hernández R., Fernández C., Baptista P. (1998). Metodología de la Investigación.

[61] Smith R.E., Smoll F.L., Cumming S.P., Grossbard J.R. (2006). Measurement of multidimensional sport performance anxiety in children and adults: The sport anxiety scale-2. J. Sport Exerc. Psychol..

[62] Otero J.M., Luengo A., Romero F., Gómez J.A., Castro C. (1998). Psicología de la Personalidad. Manual de Prácticas.

[63] Schaufeli W., Bakker A. (2003). Utrecht Work Engagement Scale.

[64] Notario-Pacheco B., Solera M., Serrano M.D., Bartolomé R., García-Campayo J., Martínez-Vizcaíno V. (2011). Reliability and validity of the Spanish version of the 10 item Connor -Davidson Resilience Scale (10 item CDRISC) in young adults. Health Qual. Life Outcomes.

[65] De García A.J., Marín M., Bohórquez M. (2012). Autoestima como variable psicosocial predictora de la actividad física en personas mayores. Revista de Psicología del Deporte.

[66] Perdomo S. (2014). Relaciones Entre Resiliencia, Apoyo Social, Estrés, Ansiedad y Depresión Sobre la Calidad de vida de Cuidadores Informales de Personas con Alzheimer. Bachelor’s Thesis.

[67] Suárez L.A. (2018). Optimismo y ansiedad en estudiantes de una Universidad Estatal de Lima. Universidad Nacional Federico Villareal. Bachelor’s Thesis.

[68] Gil C., Fetecua M., Espinosa J.C. (2017). Resiliencia y Engagement en Trabajadores de una Unidad de Cuidado Intensivo.

[69] García-León M.-A., González-Gómez A., Robles-Ortega H., Padilla J.-L., Peralta-Ramírez M.-I. (2019). Propiedades psicométricas de la escala de resiliencia de Connor y Davidson (CD-RISC) en población española. Anales de Psicología.

[70] Benetti C., Kambouropoulos N. (2006). Affect-regulated indirect effects of trait anxiety and trait resilience on self-esteem. Pers. Individ. Differ..

[71] Beutel M.E., Glaesmer H., Wiltink J., Marian H., Brähler E. (2010). Life satisfaction, anxiety, depression and resilience across the life span of men. Aging Male.

[72] Azar D., Ball K., Salmon J., Cleland V. (2008). The association between physical activity and depressive symptoms in young women: A review. Ment. Health Phys. Act..

[73] Maury-Ortiz J.G., Martínez-Lugo M.E., González-Colón Z.L. (2015). Relación del optimismo, la personalidad resistente y el engagement con el trabajo en una muestra de empleados. Revista Puertorriqueña de Psicología.

[74] Olmedilla A., Ortega E. (2012). Práctica de la actividad física y ansiedad en mujeres: Variables sociodemográficas como factores moderadores. Revista Argentina de Clínica Psicológica.

[75] McAuley E., Marquez D.X., Jerome G.J., Blissmer B., Katula J. (2002). Physical activity and physique anxiety in older adults: Fitness, and efficacy influences. Aging Ment. Health.

[76] García-Molina V.A., Carbonell-Baeza A., Delgado-Fernández M. (2010). Beneficios de la actividad física en personas mayores. Revista Internacional de Medicina y Ciencias de la Actividad Física y el Deporte.

[77] Guillén F., Angulo J. (2016). Análisis de rasgos de personalidad positiva y bienestar psicológico en personas mayores practicantes de ejercicio vs. no practicantes. Revista Iberoamericana de Psicología del Ejercicio y el Deporte.

[78] Andrade F., Pizarro J.P. (2007). Beneficio de la actividad física en el adulto mayor. Programa de diplomado en salud pública y salud familiar. Bachelor’s Thesis.

[79] Pavez P., Mena L., Vera-Villarroel P. (2012). El rol de la felicidad y el optimismo como factor protector de la ansiedad. Univ. Psychol..

[80] Vivaldi F., Barra E. (2012). Bienestar psicológico, apoyo social percibido y percepción de salud en adultos mayores. Ter. Psicol..

[81] Jürschik P., Botigué T., Nuin C., Lavedán A. (2013). Estado de ánimo caracterizado por soledad y tristeza: Factores relacionados en personas mayores. Gerokomos.

[82] Losada A.V., Álvarez M. (2014). Síntomas depresivos en adultos mayores de 65 años. Influencia del vivir solo o acompañado. Neurama Revista Electrónica de Psicogerontología.

[83] Cruz M.R. (2015). Papel de la Resiliencia en Personas Mayores Institucionalizadas. Master’s Thesis.

[84] Caicedo B., Berbesi D. (2015). Salud autorreferida: Influencia de la pobreza y la desigualdad del área de residencia. Gac. Sanit..

[85] Alomoto M., Calero S., Vaca M.R. (2018). Intervención con actividad físico-recreativa para la ansiedad y la depresión en el adulto mayor. Revista Cubana de Investigaciones Biomédicas.

[86] Cárdenas J.A., López D.A.L. (2011). Resiliencia en la vejez. Revista de Salud Pública.

[87] Vizoso C.M. (2019). Resiliencia, optimismo y estrategias de afrontamiento en estudiantes de Ciencias de la Educación. Psychol. Soc. Educ..

